# Density control method for compression test of compacted lime-flyash stabilised fiber-soil mixtures

**DOI:** 10.1016/j.mex.2018.04.010

**Published:** 2018-04-21

**Authors:** Innocent Kafodya, F Okonta

**Affiliations:** Civil Engineering Science Department, University of Johannesburg, P.O Box 524, Auckland Park, 2006, South Africa

**Keywords:** MDD, maximum dry density, OMC, optimum moisture content, UCS, unconfined compressive strength, CV, coefficient of variation, Standard test methods for laboratory compaction characteristics of soil using standard effort, West Conshohocken, PA, 2012, Standard test method for unconfined compressive strength of compacted soil-lime mixtures, West Conshohocken, PA, 200, Dry density, Target density, Specimen, Compaction, Soil composite

## Abstract

The unconfined compressive strength test is the widely accepted protocol to investigate the strength properties of lime amended soil. As a quality control measure, specimens for unconfined compression test are prepared at a predetermined maximum dry density (MDD) using standard Proctor test. Replicating MDD in a small sized mould is difficult and subject to errors, which normally arise due to inconsistent compaction efforts. The conventional method for preparing specimens involves driving a core sampler into the compacted soil to extract the specimen. The approach proves to be laborious and is associated with high material usage, as such is not ideal for investigations of many variables. To address these challenges, an alternative protocol for specimen preparation at a controlled dry density was devised. In this study, a statistical analysis of the density values was used to validate the method. The regression analysis was employed to calibrate the compaction effort for a specified target density. The method offers manifold benefits such as;

• Improved quality of specimens.
• Reduced variability of UCS test data.
• Efficiency.
• Reduced material usage.

## Graphical abstract

**Figure:**
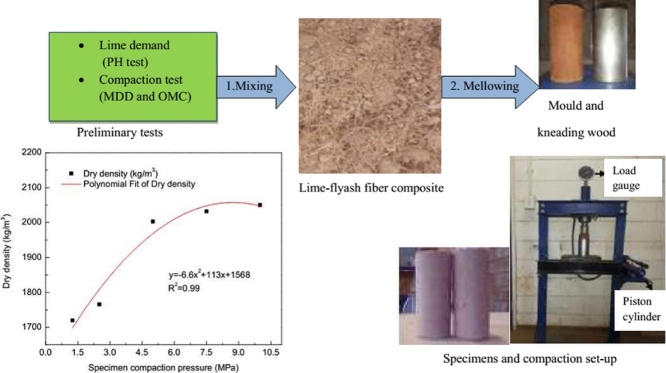

Specifications table

**Table:** 

Subject area	• *Engineering*
More specific subject area	• *Civil Engineering, Geotechnical*
Method name	• *Standard Test Methods for Laboratory Compaction Characteristics of Soil Using Standard Effort, West Conshohocken, PA, 2012.*
	• *Standard Test Method for Unconfined Compressive Strength of Compacted Soil-Lime Mixtures, West Conshohocken, PA, 200*
Name and reference of original method	• *ASTM D698*
	• *ASTM D5102*
Resource availability	• *Gauge mounted hydraulic jack*

## Method details

### Preliminary sample preparations

**Fig. 1:**
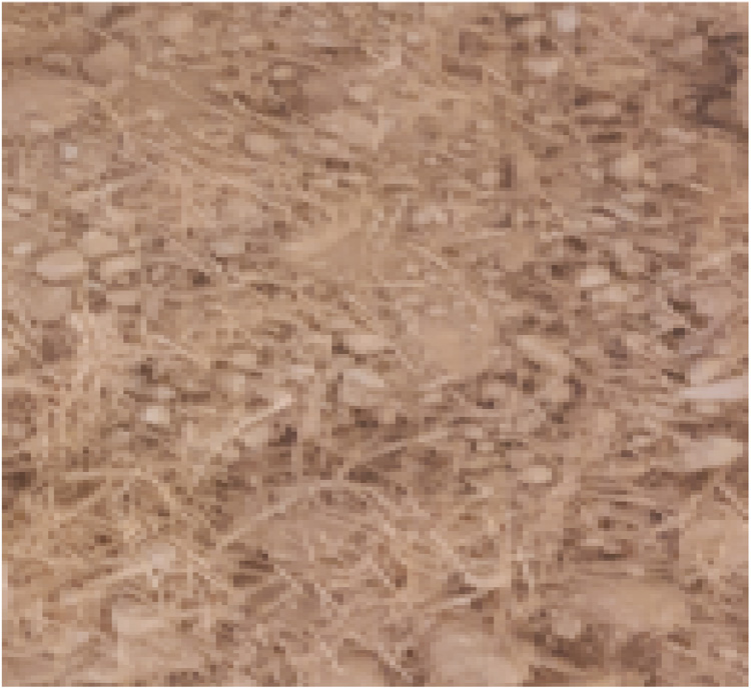
Fiber-soil composite at OMC.

The soil was initially characterised in accordance with ASTM D1140-17 [1] in order to ascertain its suitability for lime stabilisation. Maximum dry density and optimum moisture content (OMC) of the soil were determined using standard Proctor test according to ASTM D698.A commercially available hydrated lime Ca(HO)_2_ and class F flyash were used as stabilising agents. Sisal fibers were used as reinforcing elements at the content of 0.75%. The minimum lime demand for the soil was approximated by Eades-Grim test according to ASTMD 6276 [2]. The mix design for stabilising agents was 1:2 of lime and fly ash respectively according to [3].The fiber-soil composite was subsequently prepared by manually mixing lime-flyash treated soil with fibers at the optimum moisture content until homogeneous composite was formed. The fiber dosage was determined by Eq. (1).

(1) $$\rho =\frac{m_{f}}{m_{s}}$$

where $m_{f}$ is total mass of fibers and $m_{s}$ is mass of the stabilised soil. The fiber-soil mixing protocol was adapted from [4,5]. The soil composite so formed is shown in Fig. 1.

The mixture of the soil composite was allowed to mellow for 1hr according to [6] in a waterproof and airtight plastic bag prior to specimen fabrication. Mellowing allowed the soil flocculation process to reach equilibrium state.

### Specimen fabrication set-up

**Fig. 2:**
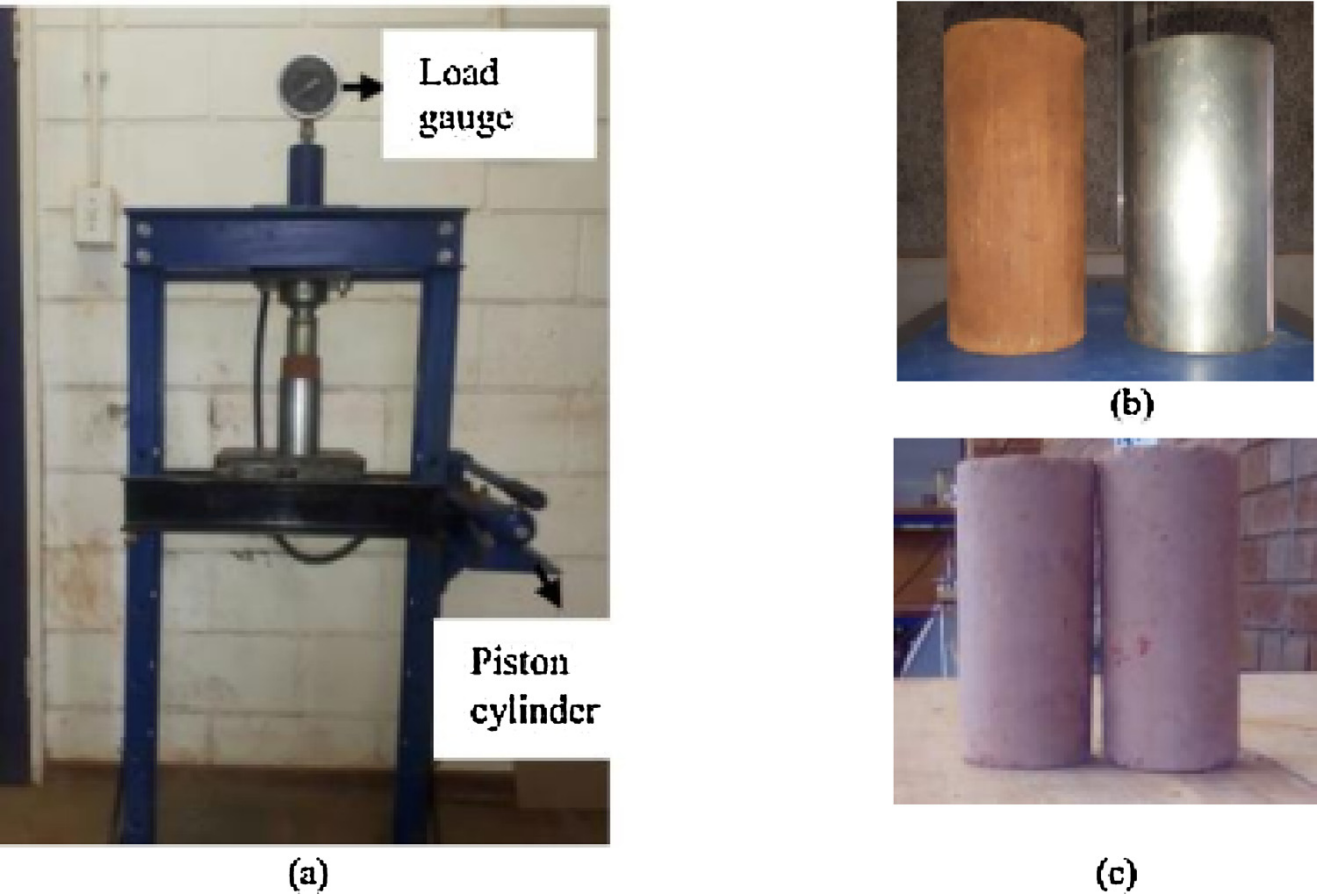
(a) Specimens compaction set-up (b) mould and kneading wood (c) fabricated specimens.

A special hydraulic jack system was devised for compacting stabilised soil composite sample (see Fig. 2a).The applied load was monitored by a load gauge mounted on the frame. The jack components and specifications are summarised in Table 1.

**Table 1:** Specifications of hydraulic jack components.

Component	Specification
Frame dimensions (bxh)	600 mm × 1500 mm
Ram diameter	50 mm
Load gauge capacity	20tons
Fluid cylinder diameter	60 mm
Fluid pipe diameter	15 mm

The mould size used herein was 55 mm diameter and 120 mm height (see Fig. 2b), and was selected to economise material usage while abiding by the recommendations in accordance with ASTM5102 procedure A [6]. Prior to compaction, the interior surface of the mould was smeared with oil to reduce friction effects. The specimens were prepared by statically compacting the soil composite into 3 layers. A wooden block of dimensions 50 mm diameter and 120 mm height (see Fig. 2b) was used to exert pressure on the soil layer. The top surface of each layer was ripped and scarified before adding a succeeding layer in order to create a continuous mass of the composite. The top surface of the specimen was eventually levelled before extrusion.

**Fig. 3:**
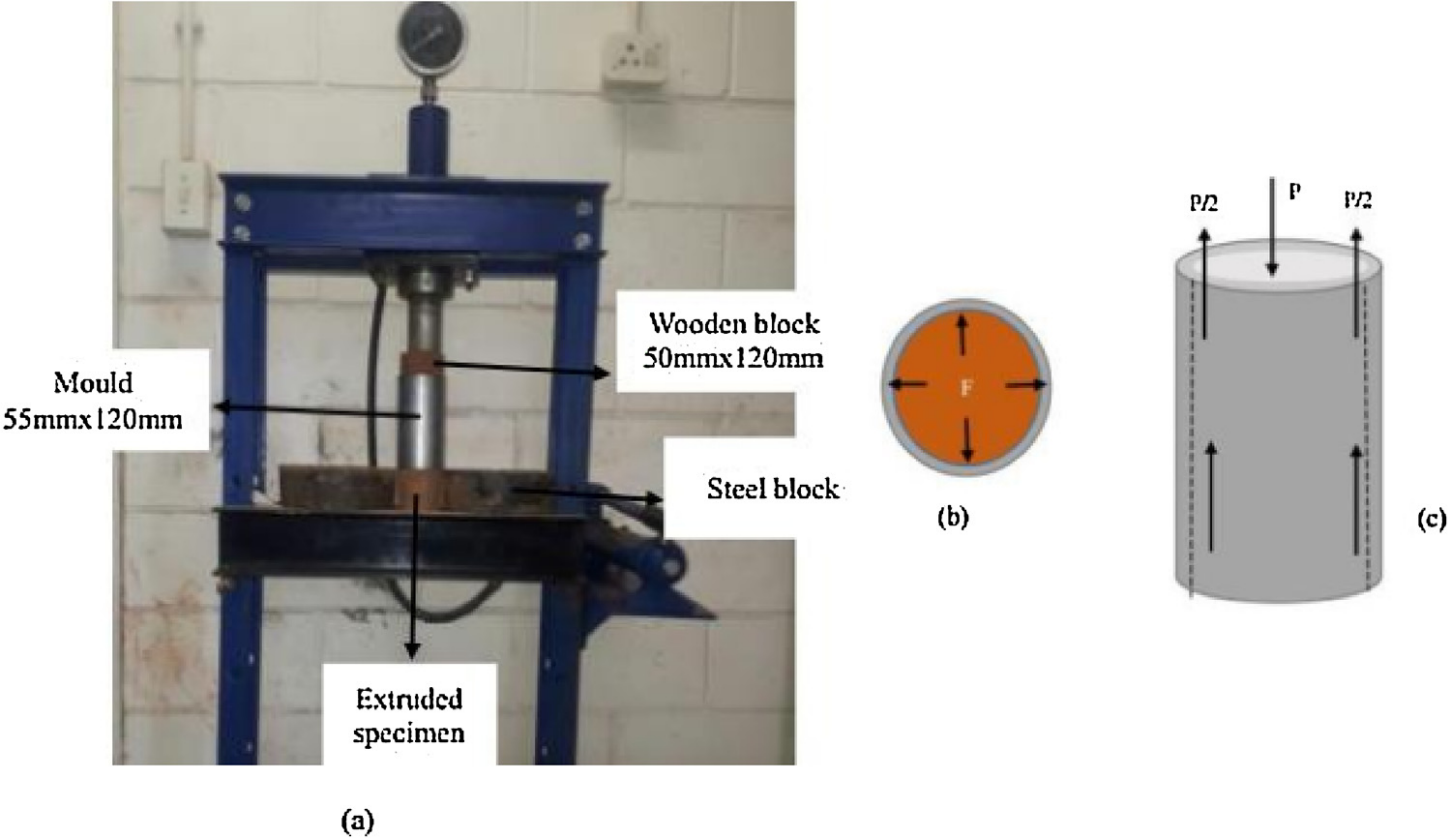
(a) Specimens extrusion set-up (b) hoop force after compaction (c) force system during extrusion.

The specimen extrusion set-up is shown in Fig. 3. In this study, specimens were extruded by applying load onto the top surface of the moulded soil. The specimen was allowed to freely move out of the mould at a constant displacement of the ram. Great care was taken to ensure that the mould’s support system could not interfere with the motion of specimen.

During compaction, deformation of the soil exerted hoop force F perpendicular to the interior surface of the mould. The hoop force F = P for equilibrium state after plastic deformation of the soil. The reactive force of the mould balanced the hoop force at the end of the compaction. At the incipient of specimen movement (refer Fig. 3c), static frictional resistance of the specimen could be expressed by Eq. (2)

(2) P=μF

where P is maximum load to initiate rigid body motion at the first stroke, F is hoop force and μ is the coefficient of friction/adhesion. In this case, the subsequent strokes were subjected to lower static frictional resistance due to reduced coefficient of friction and adhesion between soil and the mould. Sliding resistance diminished with increase in number of strokes. Greasing the interior surface facilitated sliding of the specimen. Hence, extrusion could not exert stress higher than compaction pressure that could affect soil stress state.

### Method calibration for quality control

The quality control aspects ensure that the fabricated specimens possess homogeneous properties. In respect of fiber-soil composites, uniformly distributed macrostructure and mass continuity are the fundamental parameters that dictate mechanical performance of the tested specimens. The variation of test results can be significantly minimised if great care is taken when preparing the specimens. Albeit, ASTM5102 procedure B stipulates that specimens for unconfined compressive strength test should be prepared by adapting ASTM D 698, extraction of specimens in the protocol is slow and labour intensive. Besides, material usage is high for experiments with several variables. In addition, human errors in counting number of blows (for 4.5 kg/2.5 kg rammers) lead to over compaction or under compaction of the sample. In particular, quite unlike standard compaction, this process cannot be automated.

**Fig. 4:**
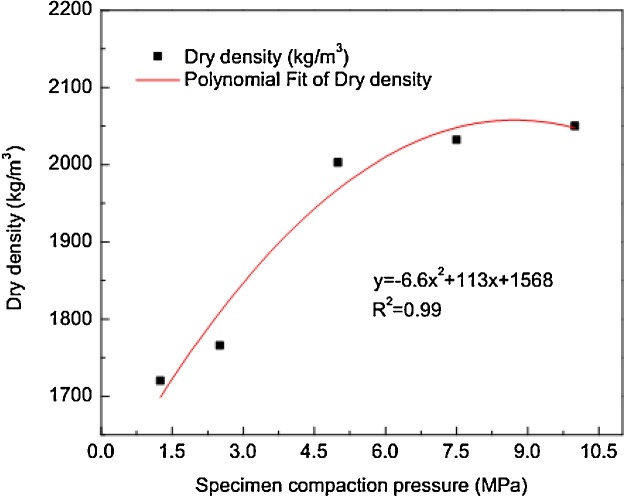
Dry density-compaction pressure relationship.

In this study, variation in compaction effort for soil layers was controlled by load gauge. However, it was imperative to determine required load level in order to achieve target dry density as obtained from the preliminary standard Proctor compaction test. To effectively estimate compaction load, regression analysis between dry density and compaction pressure was employed. The trial specimens were prepared with compaction pressures of 1.5 MPa, 3 MPa, 5 MPa 7.5 MPa and 10 MPa at the constant OMC obtained from the preliminary standard Proctor test. The dry density of the specimens at specific compaction pressure was then computed. The compaction pressure and dry density were computed using Eqs. (3) and (4) respectively.

(3) $$\sigma =\frac{12.5x10^{3}P}{D^{2}}$$

where P is the load gauge reading in tons and D is the contact surface area in (mm^2^) of the soil composite.

(4) $$\rho =\frac{100\rho_{b}}{100+w}$$

where $\rho_{b}$ is bulk density and w is OMC. The relationship and corresponding regression equation are shown in Fig. 4.

It can be seen that the required compaction pressure can be estimated by the model in Fig. 4 provided the target density is known. The coefficient of determination (R^2^) shows that 99% of the data variability from 1700 kg/m^3^ to 2040 kg/m^3^can be well explained by the model.

### Method validation

**Fig. 5:**
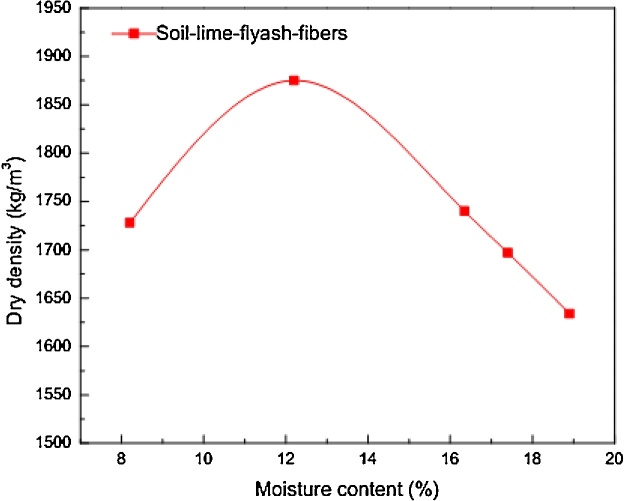
Target MDD for specimens according to ASTM D698.

The validation of the quality control method was carried out by utilising the regression model with known dry density obtained from standard Proctor test.The preliminary compaction test results of the soil-lime-flyash mixture are shown in Fig. 5.

It is shown that the maximum dry density of the mixture is approximately 1870 kg/m^3^.To reproduce this density under static compaction, the compaction effort was determined. Therefore, regression model (see Fig. 4) was used to estimate compaction pressure required to achieve the target density. For the MDD of 1870 kg/m^3^,corresponding compaction pressure was estimated as 4 MPa.The specimens were therefore prepared at 12% OMC (see Fig. 5) and every layer was compacted with 4 MPa.Specimens were prepared randomly to assess variability of densities at 4 MPa pressure.The dry density values for 70 specimens of soil-fiber, lime and flyash compositewere recorded. The statistical parameters and density values $\rho_{x}\geq \rho_{t}$ and $\rho_{x}<\rho_{t}$,where $\rho_{t}$ is target density, are shown Table 2.

**Table 2:** Statistical parameters of density data.

Parameter	Value
Mean density (kg/m^3^)	1854.88
Coefficient of variation CV	0.029
Standard deviation	54.4
$\rho_{x}\geq \rho_{t}$	30
$\rho_{x}<\rho_{t}$	40
$\rho_{t}(1-CV)\leq \rho_{x}<\rho_{t}(CV+1)$	44

$\rho_{t}$ is target dry density = 1870 kg/m^3^.

$\rho_{x}$ is number of ith density values.

**Fig. 6:**
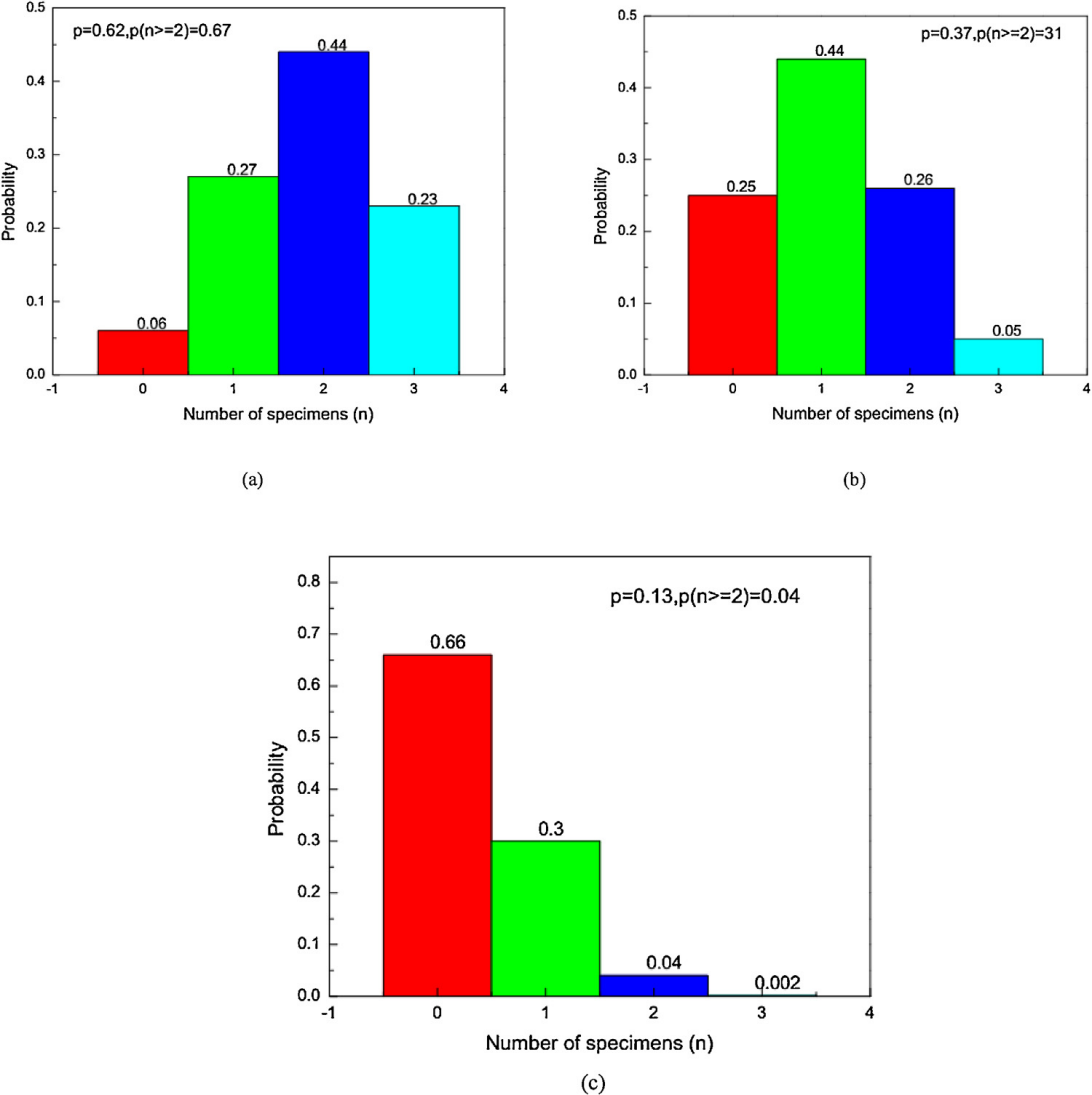
Binomial distribution graphs (a) ±3%deviation (b) ±2%deviation (c) ±1%deviation.

It should be acknowledged that the macrostructure of residual soils is composed of particles of varying sizes and specific gravity, as such any batch of soil is classified as course or fine based on the amount of dominant particle size. The specific gravity is the fundamental parameter that dictates density of the compacted soil [7]. To cater for variability in specific gravity of individual particles of fiber-lime-flyash amended soil, the probabilistic approach was employed by utilising coefficient of variation of recorded densities. The range of density values shown in Eq. (5) was considered as the most acceptable to address effects of varying particle specific gravity.

(5) $$\rho_{t}(1-CV)\leq \rho_{x}i<\rho_{t}(CV+1)$$

where $\rho_{t}$ is target dry density, $\rho_{x}i$ is density of ith specimen, CV is coefficient of variation. Since density values are random variables and that for any specimen, density could fall either below or above target value, the binomial distribution was used to determine probability of success in obtaining density value within the specified range. Using recorded dry density data, the upper and lower limits of possible density values were 1924.5 kg/m^3^and 1815.5 kg/m^3^respectively. Therefore, probability of success $P(x_{i})=p$ was 0.62 and, the corresponding probability of failure was 0.38.The density range converged towards target density with the decrease in CV. Varying CV to1.9% and 0.9% led to the probability of success equal to 0.37 and 0.13 respectively and corresponding probability of failure of 0.63 and 0.87 respectively. The binomial distribution model shown in Eq. (6) was used to plot the probability distribution graphs.

(6) $$P(x,n,p)=\frac{n!p^{x}{\left(1-p\right)}^{n-x}}{x!(n-x)!}$$

where n is the number of fabricated specimens, x is the number of successes and p is probability of obtaining density within the range. The laboratory procedure recommends a minimum of 2 specimens for a set of UCS specimens of lime amended soil [6]. If 3 specimens are prepared, the binomial distribution graphs for the above highlighted probability scenarios are shown in Fig. 6a–c

It is seen that for a minimum of 3 specimens, the acceptable density values of specimens should be within ±3% target density for atleast 2 specimens to fall within the range.

### Method optimisation

The forgone section has shown that for a given set of density data, values converge towards target density when the deviation reduces. Therefore, it is imperative to minimise the deviation of the data from the target value while utilising few specimens in order to save time and materials. Binomial probability distribution could be used to optimise the number of specimen and deviation. This could be achieved by formulating objective functions and associated constraints. In this case, two fundamental objective functions were formulated as shown in Eqs. (7) and (8) subject to Eq. (9).

(7) $$\text{Max}:P(x,n,p)=\frac{n!p^{x}{\left(1-p\right)}^{n-x}}{x!(n-x)!}$$

(8) $$\text{Min}:\left(n,\rho_{t}(1-CV)\leq \rho_{x}i<\rho_{t}(CV+1)\right)$$

(9) Subject to constraints:P(x,n,p)≥50% and 2≤n≤5

If coefficient of variation is taken as maximum acceptable deviation, then conditions in Eq. (10) and denoting CV = β give;

(10) $$\lim_{\beta \to 0}\rho_{t}(1-\beta )\leq \rho_{xi}<(1+\beta )\rho_{t}$$

**Fig. 7:**
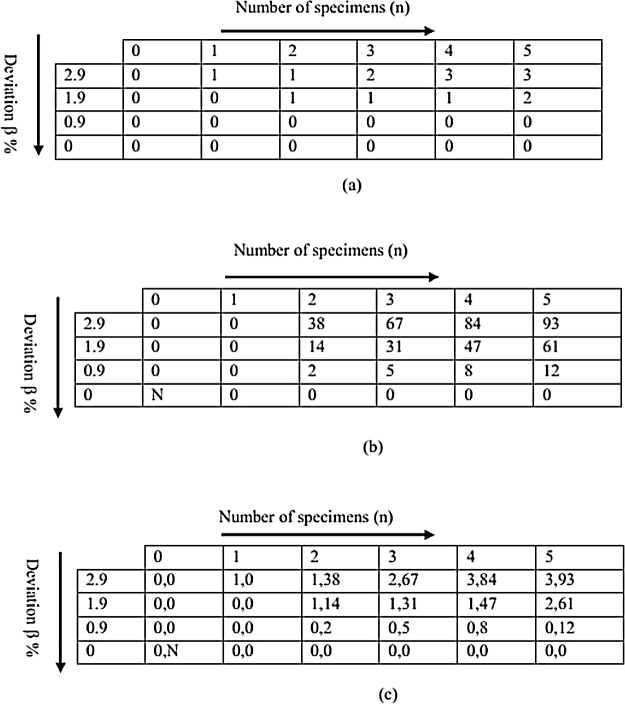
Probability matrices (a) possible number of specimens to fall within acceptable range (b) atleast 2 specimens fall within acceptable range (c) combination of a and b matrices.

For β = 2.9%,1.9%, 0.9% and 0%,the 4 × 5 matrix could be generated with elements representing likelihood and probability of success of a given number of specimens (n,P(x*,n,*p).Figs. 7a–c show matrices of the most likely number of specimens for a given deviation and the corresponding probability of falling within acceptable range.

The above matrices imply that if 3 specimens are prepared, it is likely to get 2 or more density values within approximately ± 3% deviation from the target density. When the acceptable deviation range is reduced to approximately ± 2%, the minimum number of specimens should be 5.On the basis of material usage and time, 3 is the optimal number of specimens.

## Rationale behind the method

The conventional method to test strength properties of lime stabilised soil is by conducting a series of unconfined compressive strength tests of the cured soil as stipulated by ASTM5102 [6,[8], [9], [10]].The dry density and moisture content of soil during specimens’ preparation are key parameters that dictate the mechanical properties of the soil. In the UCS test, controlling dry density of specimens improves the quality of the data. The density can be controlled by establishing target density which is the MDD of the soil. The MDD is determined by the standard Proctor compaction test of the soil according to ASTM D698 [11].To replicate the MDD for small sized specimens is a challenging task. However, conventional approach is to drive core sampler into the compacted soil which is labourious and time consuming or adapting ASTM5102 procedure A, the latter being subject to human errors. Compacting soil in the small moulds of 1:2.5 ratio of diameter to height proves difficult to achieve desired dry density. However, the moulds can be used if the required compaction effort for the desired density is established. To establish required compaction effort for small mould is a jigsaw puzzle that requires accurate relationship of compaction effort and density to be determined. It is necessary therefore to apply constant effort in order to achieve dry density within acceptable range closer to the desired density for a set of UCS specimens. This can be achieved by monitoring compaction pressure using load gauge. This load controlled method was adapted in our previous research to investigate the effects of pre-compression and natural fibers inclusions on the UCS of the lime-fly ash stabilised soil. The effective application of natural materials in civil engineering is the backbone of sustainable infrastructure development.

## Concluding remarks

The analysis of the proposed method indicates that;

• The method is more practical to replicate MDD in a small mould of diameter to height ratio of 1:2.5.
• The compaction pressure of any given soil must be well calibrated in order to achieve desired density.
• For every 3 specimens, at least 2 will fall within ± 3% of the target density
• For a set of at least 3 specimens, it is very likely that 3 specimens will fall within ± 3% of the target density.
• When acceptable density range is reduced to ± 2%, a minimum of 5 specimens is recommended.
• On the basis of minimising material usage and time,3 specimens are recommended.
