# Supplementary material for: Density control method for compression test of compacted lime-flyash stabilised fiber-soil mixtures
Source: MethodsX. 2018 Apr 21;5:848–56. doi: 10.1016/j.mex.2018.04.010 (PMC6097468; doi:10.1016/j.mex.2018.04.010)
Supplement: Supplementary file 1 [file mmc1.docx]

Supplementary materials

Recorded specimens’ density data

| Specimen serial | Mass  g | Bulk density  kg/m^3^ | Dry density  kg/m^3^ |
| --- | --- | --- | --- |
| 1  2  3  4  5  6  7  8  9  10  11  12  13  14  15  16  17  18  19  20  21  22  23  24  25  26  27  28  29  30  31  32  33  34  35  36  37  38  39  40  41  42  43  44  45  46  47  48  49  50  51  52  53  54  55  56  57  58  59  60  61  62  63  64  65  66  67  68  69  70 | 589.5  566  591  575  600  591.5  564  568.5  609.5  559.5  589.5  592.5  578  587.5  563.5  574.5  576  582  561.5  568  581.5  581  581.5  568.5  581.5  587.5  590.5  575  567  599.5  584.5  611  594.5  568.5  565  563.5  592.5  594  611.5  620.5  616  606  609.5  617  614.5  615.5  605  616  616  610.5  606.5  621.5  610.5  606  609.5  605.5  605  607.5  605  622.5  590.5  608  601.5  600.5  585  578  590  589.5  600  585 | 2067.7  1985.27  2072.96  2016.84  2104.53  2074.71  1978.26  1994.04  2137.85  1962.47  2067.7  2078.22  2027.36  2060.68  1976.50  2015.09  2020.35  2041.39  1969.49  1992.29  2039.64  2037.88  2039.65  1994.04  2039.64  2060.68  2071.20  2016.83  1988.77  2102.77  2050.16  2143.11  2085.23  1994.04  1981.76  1976.50  2078.22  2083.48  2144.86  2176.43  2160.64  2125.57  2137.84  2164.15  2155.38  2158.89  2122.06  2160.64  2160.64  2141.35  2127.32  2179.94  2141.35  2125.57  2137.84  2123.81  2122.06  2130.83  2122.06  2183.44  2071.20  2132.58  2109.78  2106.28  2051.91  2027.36  2069.45  2067.69  2104.52  2051.91 | 1846.15  1772.56  1850.85  1800.74  1879.04  1852.42  1766.30  1780.39  1908.79  1752.20  1846.15  1855.55  1810.14  1839.89  1764.73  1799.18  1803.88  1822.67  1758.47  1778.82  1821.10  1819.53  1821.10  1780.39  1821.10  1839.89  1849.29  1800.74  1775.69  1877.47  1830.50  1913.49  1861.81  1780.39  1769.43  1764.73  1855.55  1860.25  1915.05  1943.24  1929.15  1897.83  1908.79  1932.28  1924.45  1927.58  1894.70  1929.15  1929.15  1911.92  1899.39  1946.37  1911.92  1897.83  1908.79  1896.26  1894.70  1902.53  1894.70  1949.50  1849.29  1904.09  1883.74  1880.60  1832.06  1810.14  1847.72  1846.15  1879.04  1832.066 |
